# Significance of m^6^A regulatory factor in gene expression and immune function of osteoarthritis

**DOI:** 10.3389/fphys.2022.918270

**Published:** 2022-09-08

**Authors:** Xiaoyan Xie, Yun Zhang, Jian Yu, Feng Jiang, Chuyan Wu

**Affiliations:** ^1^ Department of Rehabilitation Medicine, The First Affiliated Hospital of Nanjing Medical University, Nanjing, China; ^2^ Department of Neonatology, Obstetrics and Gynecology Hospital of Fudan University, Shanghai, China

**Keywords:** m^6^A regulators, osteoarthritis, random forest model, immunity, m^6^A score

## Abstract

One of the most prevalent posttranscriptional modifications of eukaryotic mRNA is the RNA N6-methyladenosine (m^6^A) regulator, which plays a significant role in various illnesses. The involvement of m^6^A regulators in osteoarthritis (OA) is not fully known. By comparing nonosteoarthritic and osteoarthritic patients, 26 important m^6^A regulators were identified from the gene expression omnibus GSE48556 dataset. Seven candidate m^6^A regulators (IGFBP3, WTAP, IGFBP1, HNRNPC, RBM15B, YTHDC1, and METTL3) were screened using a random forest model to assess the likelihood of OA. A column line graph model founded on seven m^6^A modulator candidates was created. According to decision curve analysis, patients might profit from the column line graph model. Based on chosen relevant m^6^A modifiers, a consensus clustering approach was utilized to categorize OA into two m^6^A categories (group A and group B). To measure the m^6^A pattern, a principal component analysis technique was created to generate the m^6^A score for every sample. Cluster A patients exhibited more excellent m^6^A scores than cluster B patients. Furthermore, we discovered that patients with lower and higher m^6^A scores had varied immunological responses using the m^6^A type. At last, m^6^A regulators contribute significantly to the progression of OA. Our research on m^6^A patterns might help to guide further OA immunotherapeutic techniques.

## Introduction

The most prevalent musculoskeletal illness, osteoarthritis (OA), is characterized by localized joint space restriction, osteophyte, subchondral bone sclerosis, and subchondral cysts (Ariani et al., 2019). Because of the considerable advancement in OA studies, it is generally thought that OA is a complicated degenerative disease influenced by various variables, including age, obesity, inflammation, trauma, and genetics (Yucesoy et al., 2015). In recent years, there have also been studies that bone arthritis is a low-grade inflammatory state closely linked to some inflammatory mediators. Patients with OA have high amounts of Interleukin (IL)-1, IL-10, IL-17, and tumor necrosis factor (TNF) in their blood and synovial fluid (Hussein et al., 2008). Other studies also found that the leukocytes and monocytes necessary to regulate the immune response are more marvelous and proinflammatory in OA patients, and inflammation and body mass index are related to this (Loukov et al., 2018). Therefore, early screening and effective prevention of OA patients from an immunological point of view will profoundly influence the control of OA.

N6-methyladenosine (m^6^A), 5-methylcytidine (m^5^C), N6-2′-O-dimethyladenosine (m^6^Am), pseudouridine (Ψ), 1-methyladenosine (m^1^A), and 5-hydroxymethylcytidine (hm^5^C) have all been studied extensively in latest days (Jonkhout et al., 2017). Among them, m^6^A is the most popular, abundant and well-characterized internal modification in messenger RNA (mRNA). The N6-methylation of altered RNA may influence its fold, durability, degradation, and cellular interaction(s), involving it in splicing, translation, export, and decay processes (Oerum et al., 2021). Recent studies have reported that m^6^A RNA methylation can regulate promoter–proximal pausing of RNA polymerase II, and m^6^A RNA methylation mediated in methyltransferase-like 3 (METTL3) can promote antitumor immunity in natural killer cells (Akhtar et al., 2021; Song et al., 2021). m^6^A has been shown to have a critical role in a variety of disorders, including cancer (Liu et al., 2018; Sun et al., 2019), asthma (Dai et al., 2021), osteoporosis (Wu et al., 2018), and Alzheimer’s disease (Han et al., 2020). And METTL3–mediated m^6^A modification of ATG7 regulates autophagy and can promote OA progression (Chen et al., 2022). Nevertheless, the specific role of the m^6^A regulatory factor in OA immunity is unclear.

**FIGURE 1:**
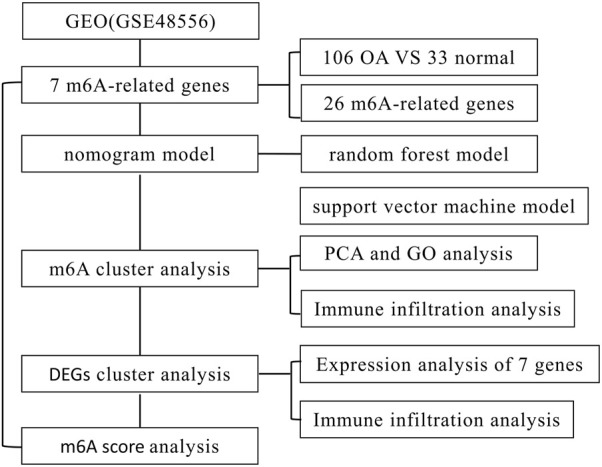
Flow chart.

This research synthesized the involvement of the m^6^A regulatory factor in the identification and subtype categorization of OA using the GSE48556 dataset from the GEO database. Based on seven candidate m^6^A regulators, we created a gene model for predicting OA susceptibility [Insulin-Like Growth Factor Binding Protein 3 (IGFBP3), Wilms tumor 1–associating protein (WTAP), heterogeneous nuclear ribonucleoproteins (HNRNPC), Insulin-Like Growth Factor Binding Protein 1 (IGFBP1), RNA-binding motif protein 15B (RBM15B), YTH domain–containing protein 1 (YTHDC1), and METTL3], and predict the prevalence of OA from the perspective of m^6^A regulation. In addition, we compared two types of immune responses, which provides a new idea for the treatment of OA (Figure 1 flowchart).

## Materials and methods

### Data gathering

The GEO database (http://www.ncbi.nlm.nih.gov/geo/) was used to pick a dataset (GSE48556) of 106 and 33 non-OA healthy adults. Through peripheral blood gene analysis between OA patients and non-OA patients, we extracted 26 m^6^A regulators (Supplementary File S1) from the dataset. The following are the criteria we use to choose datasets: first, the sample size must be larger than 100; second, the data set must include both a treatment and a healthy control; third, in both the patient and control groups, there must be at least 10 cases in each. These regulators consisted of nine writers (METTL3, METTL14, METTL16, WTAP, VIRMA, RBM15, RBM15B, CBLL1, and ZC3H13), two erasers (FTO and ALKBH5), and 15 readers (YTHDC1, YTHDC2, YTHDF1, YTHDF2, YTHDF3, HNRNPC, FMR1, LRPPRC, HNRNPA2B1, IGFBP1, IGFBP2, IGFBP3, RBMX, ELAVL1, and IGF2BP1) (Dai et al., 2021; Jiang et al., 2022).

### Creating the random forest model and the support vector machine model

As a training model, create a random forest (RF) and support vector machine (SVM) method to forecast the incidence of OA. To assess the model, plot the reverse summary statistics of residuals, boxplots of residuals, and receiver operating characteristic (ROC) curves. The RF approach is a regression tree method that permits bootstrap aggregation and predictor randomization to obtain a high level of predicted accuracy (Rigatti, 2017). In our work, an RF model was constructed using the “Random Forest” package of the R statistical program (Version 4.0.4). Potential m^6^A regulators were chosen from a pool of 26 m^6^A regulators to forecast the incidence of OA. In our investigation, ntrees and mtry were adjusted to 100 and 3, correspondingly. The relevance of 26 m^6^A regulators was then assessed, and the suitable important m^6^A regulators were chosen using 10*x* cross-validation. The *y*-axis of the 10*x* cross-validation curve correlates to the model’s accuracy when the number of m^6^A regulators is changed. SVM is a supervised machine learning method based on the statistical learning theory of structural risk reduction. In our investigation, each data point was represented as a point in *n*-dimensional space (where *n* is the number of m^6^A regulators). We then discovered an ideal hyperplane that effectively distinguishes between the two groups.

### Establishment of nomogram model

We utilized the “RMS” tool in R to forecast the occurrence of OA using a nomogram model depending on the chosen seven candidate m^6^A regulators. The calibration graph is also used to evaluate the projected value to the actual value, followed by an analysis of the decision curve and a clinical impact curve to see whether the model is helpful for illness prediction in patients (Iasonos et al., 2008).

### Recognition of m^6^A pattern

To detect distinct m^6^A patterns, the “ConsensusClusterPlus” software program in R is used (Wilkerson and Hayes, 2010), and the “limma” package in R is used to search differentially expressed genes (DEGs) between m^6^A trends. *p* < 0.01 was used as a screening criterion. The “ConsensusClusterPlus” software program in R software is used to do functional enrichment analysis to better understand the various mechanisms of DEGs involvement in OA, and the findings are shown in an enrichment circle diagram.

### Quantification of m^6^A patterns and assessment of immune cell abundance

We utilize the principal component analysis (PCA) technique to produce the m^6^A score, which is calculated as follows: PC1i = m^6^A score, where PC1 is principal component 1, and i is the DEG expression (Zhang et al., 2020). Gene set enrichment analysis (ssGSEA) is also used to evaluate the gene expression patterns in the samples. Then these genes were searched in the input data to quantify the presence of these genes in immune cells in every sample.

### Statistical analysis

Correlations among writers, erasers, and reader are discovered using linear regression analysis. Then, the residuals were tested in the linear regression via quantile–quantile plots (Q–Q plot). Kruskal–Wallis analysis is used to assess component differences. All parametric analyses are defined as two tests, with *p* < 0.05 considered statistically significant. R version 4.0.4 was used for all statistical studies.

## Results

### The landscape of the 26 m^6^A regulators in osteoarthritis

**FIGURE 2:**
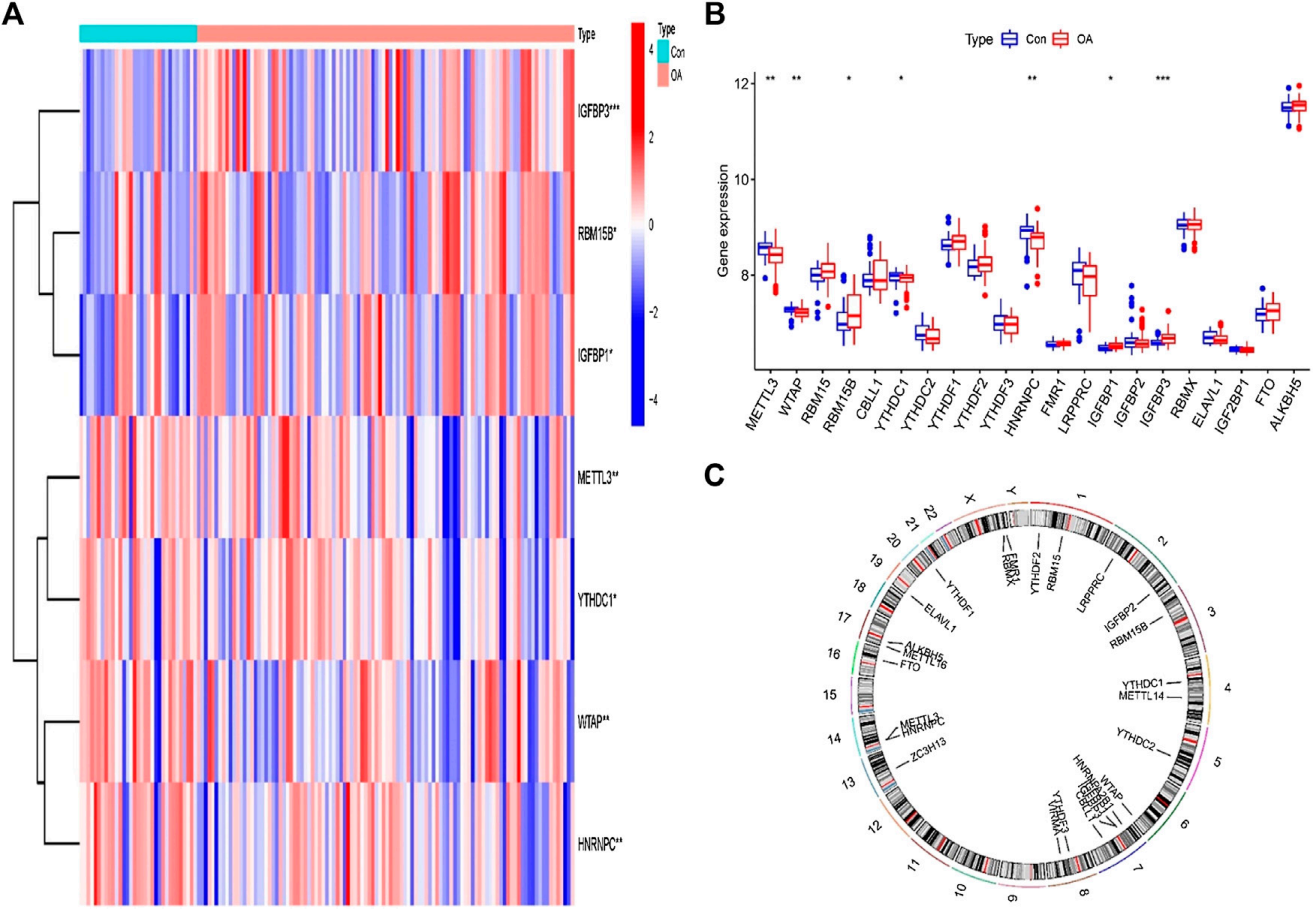
m^6^A modulators in osteoarthritis (OA). **(A)** Heat maps of the expression of seven important m^6^A regulators in patients with nonosteoarthritis and OA. **(B)** Histogram of the differences in the expression of 21 m^6^A regulators identified between subjects with nonosteoarthritis and OA. **(C)** The chromosomal position of the m^6^A regulator. *p < 0.05, **p < 0.01, and ***p < 0.001.

The R software package “limma” was used to compare the expression levels of m^6^A regulators in patients with and without OA. Seven important m^6^A regulators (IGFBP3, RBM15B, IGFBP1, METTL3, YTHDC1, HNRNPC, and WTAP) were screened and visualized using heatmaps and histograms. We discovered that RBM15, RBM15B, and YTHDF1 were abundantly expressed in OA patients, but the other critical m^6^A regulators showed lower or equivalent expression in OA cases in contrast to individual patients (Figures 2A, B). Use the “RCircos” software tool to see the chromosomal locations of m^6^A regulators (Figure 2C).

### Correlation among the writer, eraser, and readers in osteoarthritis

**FIGURE 3:**
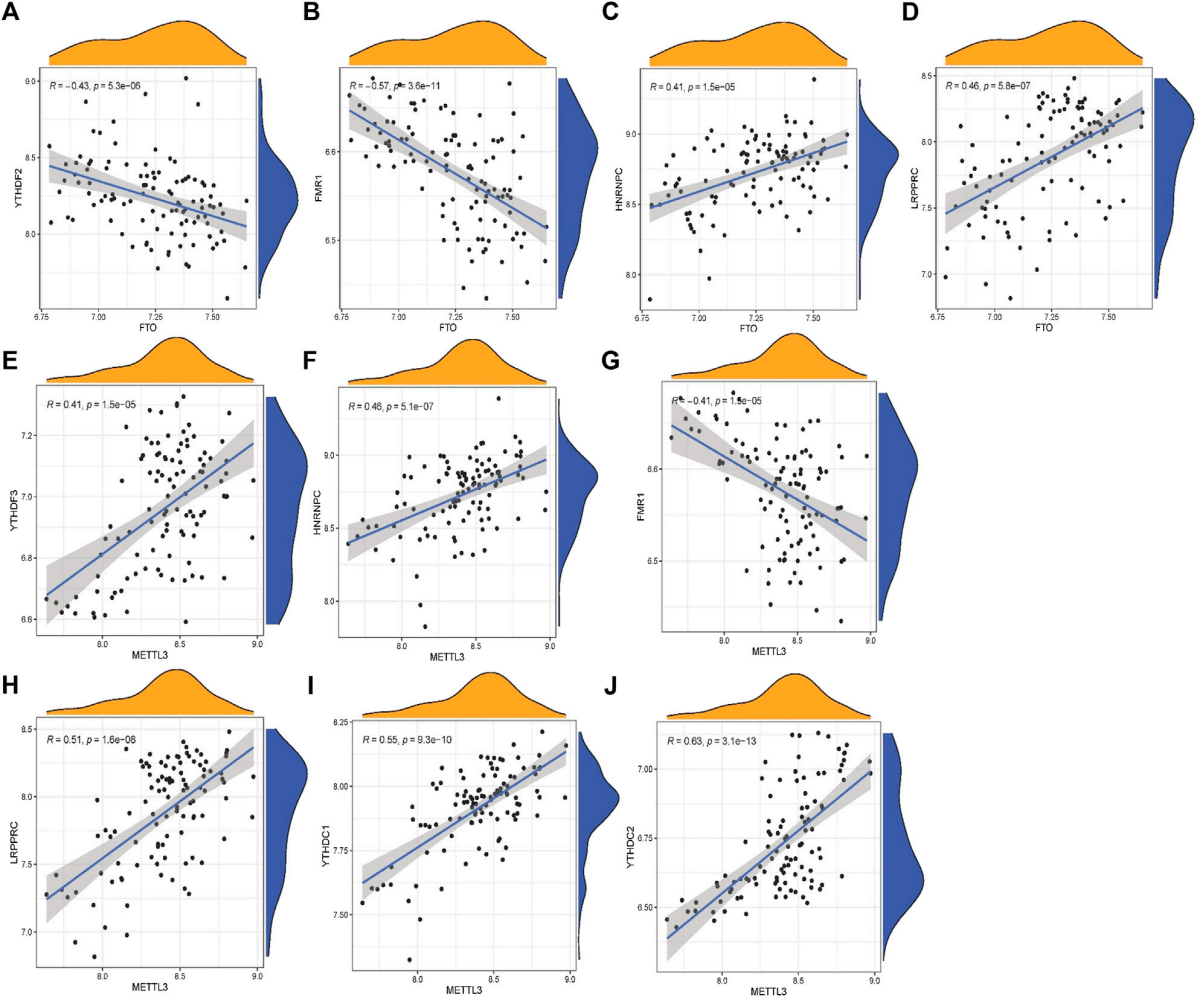
Correlation among writers, erasers, and readers in OA **(A–J)**. Reader genes: YTHDF2, FMR1, HNRNPC, LRPPRC, YTHDF3, YTHDC1, and YTHDC2; writer and eraser genes: METTL3 and FTO.

To explore the relationship between writer, eraser, and reader in OA, we used linear regression analysis to examine the correlation one to one. Then, we tested the residuals in the linear regression via Q–Q plot (Supplementary Figure S1). We found only significant differences between eraser and reader and between writer and reader. HNRNPC and LRPPRC expression levels in OA patients were substantially linked with FTO. The expression levels of YTHDF2 and FMR1 are highly negatively correlated with FTO. High expression of YTHDF3, HNRNPC, LRPPRC, YTHDC1, and YTHDC2 was positively correlated with METTL3, whereas patients with high expression of FMR1 had lower levels of METTL3 expression. Writer and eraser have no significant correlation (Figure 3).

### Creating the random forest model and support vector machine model

**FIGURE 4:**
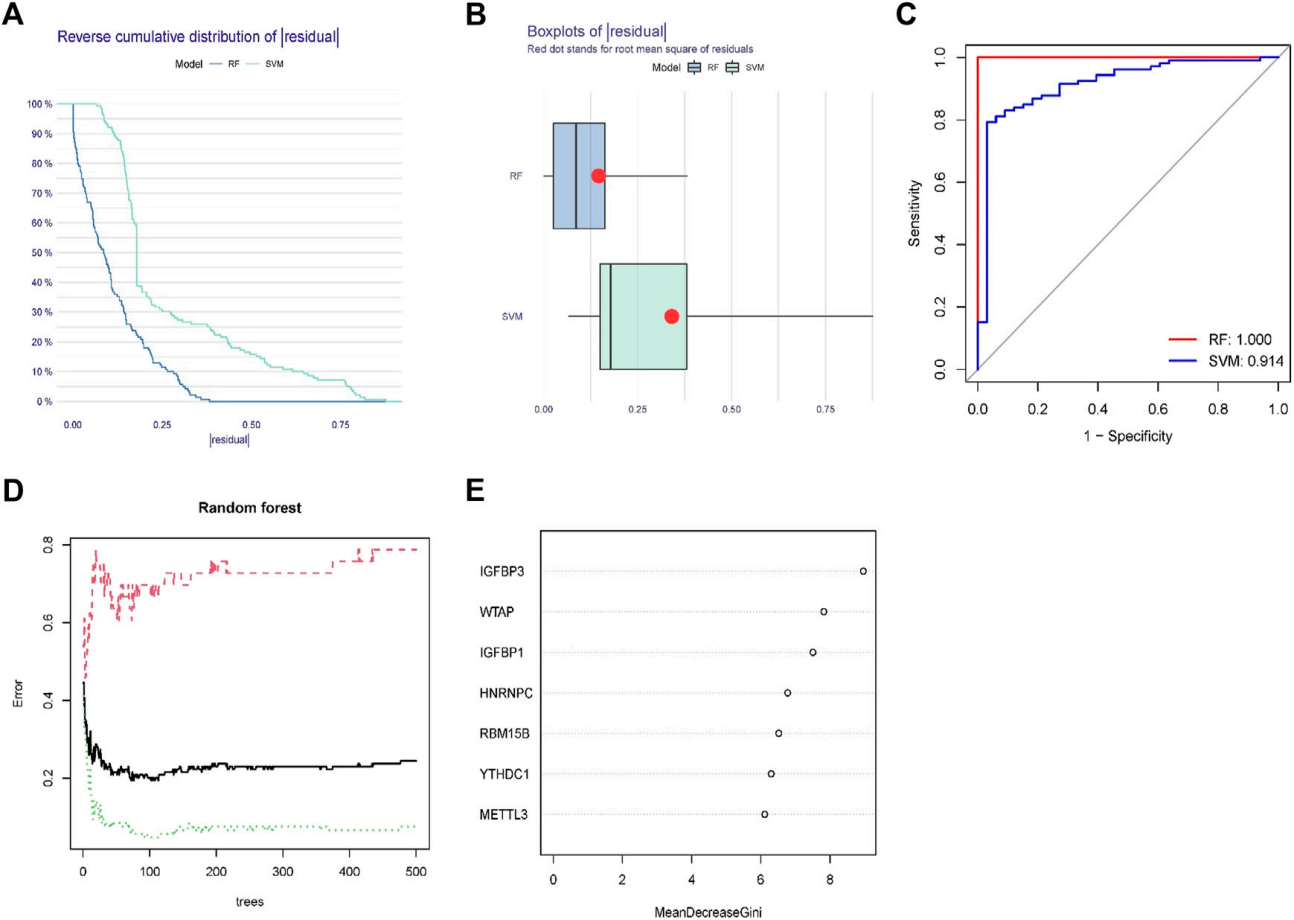
Random forest(RF) model and support vector machine model. **(A)** The residual distribution of the RF and support vector machine (SVM) models was displayed using the reverse cumulative distribution of residuals. **(B)** The residual distribution of the RF and SVM models was displayed using boxplots of residuals. **(C)** The receiver operating characteristic curves showed how accurate the RF and SVM models were. **(D)** The tree model demonstrates that the error has stabilized. **(E)** The seven m^6^A regulators’ relative relevance.

To predict the incidence of OA, we developed RF and SVM models. The RESIDUE of the RF model is negligible, according to both the “inverse cumulative distribution of residues” (Figure 4A) and the “remnant boxline plot” (Figure 4B). The majority of the samples in the model have tiny residuals, suggesting that the model is superior. As seen by the ROC curve, RF is more reliable than SVM (Figure 4C). The correlation plot between model uncertainties and the number of selection trees (Figure 4D) revealed a consistent inaccuracy; thus, we picked 500 trees as the variable of the final model. Throughout the creation of the RF model, the variable significance of the output results (Gini coefficient technique) was examined in the context of declining accuracy and reducing mean square error. Figure 4E reveals that seven m^6^A regulators (IGFBP3, WTAP, IGFBP1, HNRNPC, RBM15B, YTHDC1, and METTL3) are more important than five points.

### Construction of the nomogram model

**FIGURE 5:**
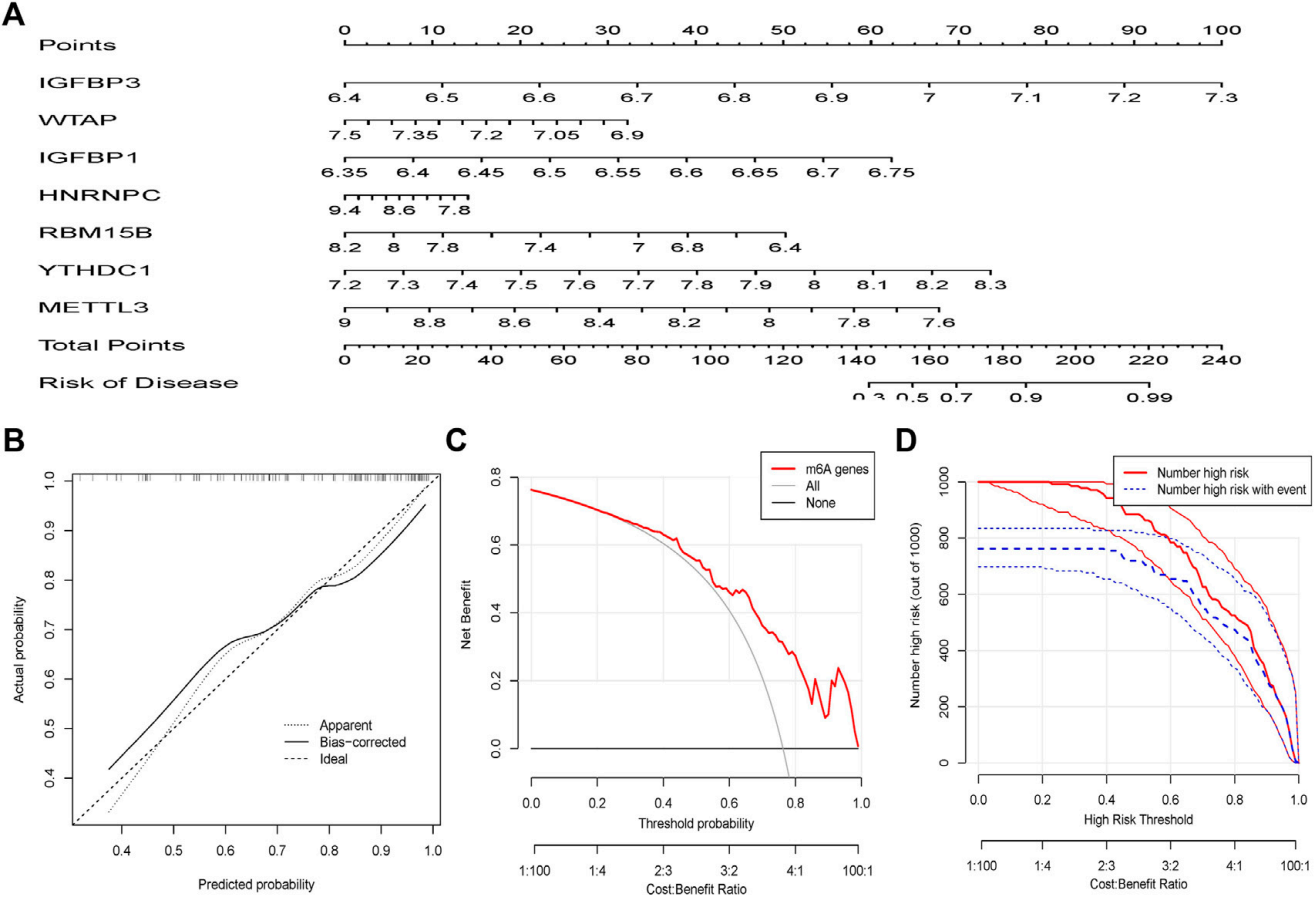
The nomogram model is created. **(A)** The nomogram model was built using the m^6^A regulators of the seven candidates. **(B)** The calibration curve reveals the nomogram model's predictive capacity. The calibration curve reveals the nomogram model’s predictive capacity. **(C)** Patients with OA may benefit from relying on the nomogram model. **(D)** The clinical relevance curve is used to analyze the nomogram model’s clinical effect.

To predict the occurrence of OA patients, a line graph model was built in R using the “rms” package against seven potential m^6^A regulators (Figure 5A). It can be seen from the prediction curve that the column chart model is accurate (Figure 5B). The red line stays above the gray and black lines between 0 and 1, as shown in the Decision Curve Analysis (DCA) curve, indicating that this column line chart model may be advantageous for patients with OA (Figure 5C). The clinical impact curve shows that the line plot model has excellent predictive power (Figure 5D).

### Two different m^6^A modes distinguished by important m^6^A regulators

**FIGURE 6:**
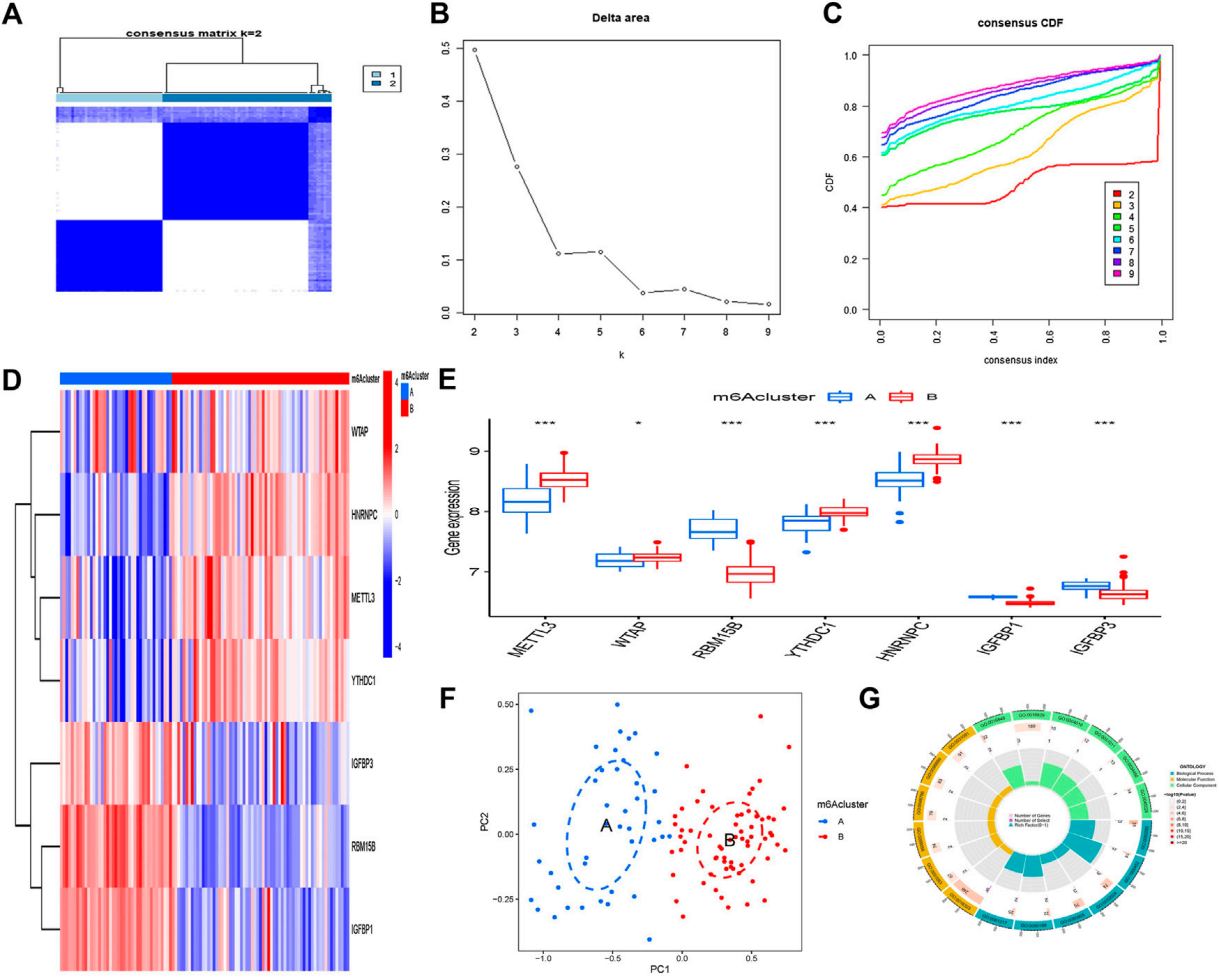
Clustering of the seven major m^6^A regulators in OA by consensus. **(A)** Matrix consensus of the seven major m^6^A regulators for *k* = 2. **(B)** The difference of delta area for *k* = 2–9. **(C)** The area under the CDF curve. **(D)** Cluster A and B expression heatmaps of the seven important m^6^A regulators. **(E)** Histogram showing differential expression of the seven major m^6^A regulators in clusters A and B. **(F)** The expression patterns of the seven key m^6^A regulators were analyzed using principal component analysis, which revealed significant differences in gene sequences between the two m^6^A patterns. **(G)** The influence of the 639 m^6^A-related differentially expressed genes (DEGs) on the onset and progression of OA is investigated using gene ontology analysis. *p < 0.05, **p < 0.01, and ***p < 0.001.

Two m^6^A patterns (A and B) were identified using the “ConsensusClusterPlus” tool of R software for the seven m^6^A regulators. The difference in delta area is seen in Figures 6A, B because the area tends to remain stationary when *k* = 2; this is the best *k*-value to use. When *k* = 2, the cluster research findings are the most trustworthy, and the resultant classification is the greatest. Figure 6C displays the average values when *k* assumes various values. Cluster A contained 41 cases, and cluster B contained 65 cases. The differential expression values of the seven key m^6^A regulators between the two groups are then shown using heatmaps and histograms (Figure 6D). RBM15B, IGFBP1, and IGFBP3 are expressed at higher levels in group A than those in group B, whereas METTL3, WTAP, YTHDC1, and HNRNPC showed the opposite (Figure 6E). The PCA shows that seven important m^6^A regulators can fully distinguish between these two m^6^A modes (Figure 6F). A total of 639 DEGs with m^6^A correlations were chosen from the two m^6^A modes (Supplementary File S2). GO functional enrichment analysis was used to investigate the likely processes of these DEGs in osteoarthritis, and the findings were shown using an enrichment circle chart (Figure 6G). These genes were mostly enriched in the GO terms 0016829 and 0016323, which were associated with lyase activity and basolateral plasma membrane, correspondingly.

**FIGURE 7:**
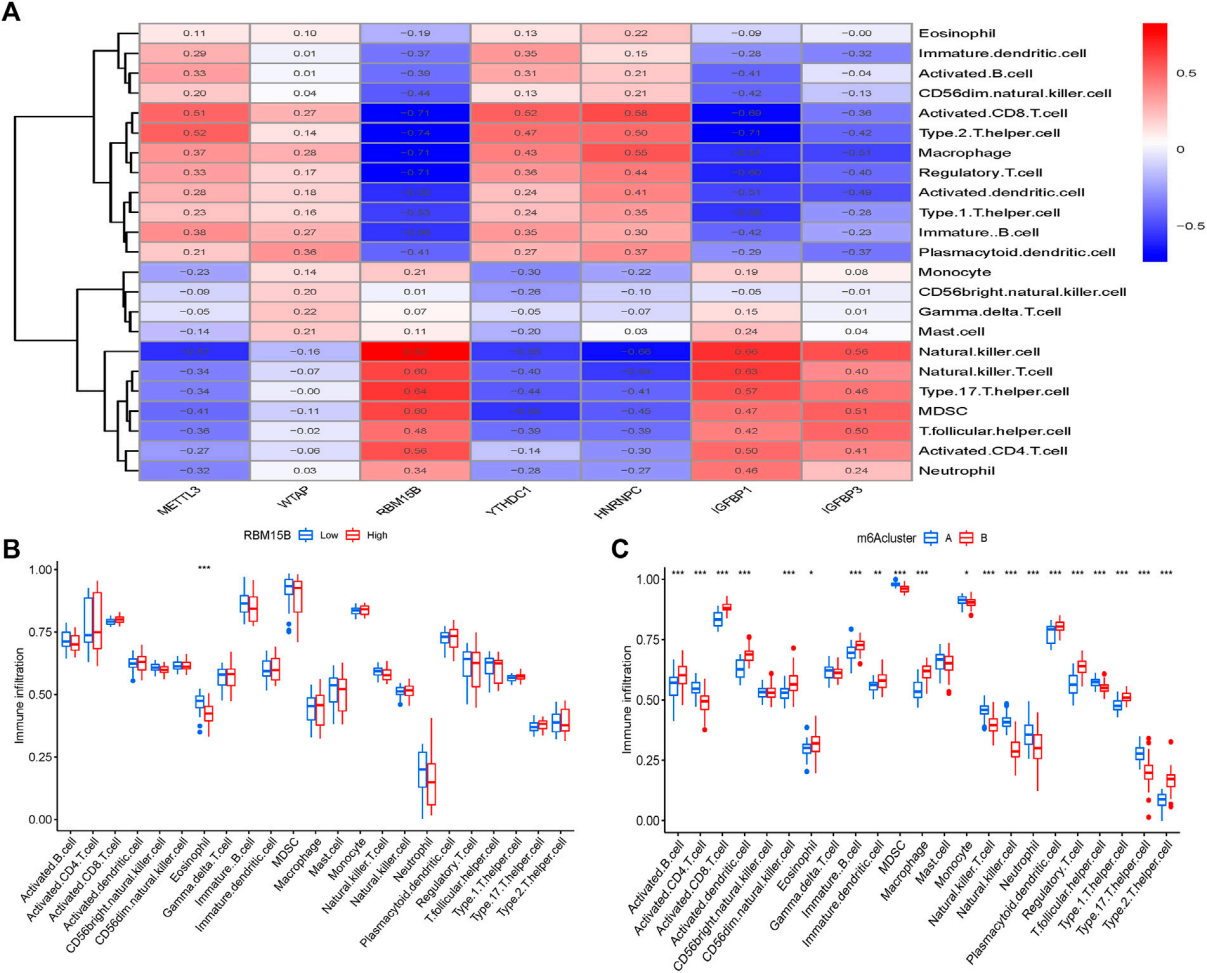
Gene collection from a single sample and enrichment analysis. **(A)** Correlation between immune cells and seven key m^6^A modulators found in different species. **(B)** Differences in the number of infiltrative immune cells between groups with lower and higher RBM15B expression. **(C)** Immune cell infiltration differences between clusters A and B. *p < 0.05, **p < 0.01, and ***p < 0.001.

The quantity of immune cells in OA samples was then calculated using ssGSEA, and the association between seven key m^6^A regulators and immune cells was assessed. We discovered that the m^6^A methyltransferase RBM15B is linked to a variety of immune cells (Figure 7A). The variations in immune cell infiltration between individuals with elevated/low RBM15B expression were investigated. Immune cell infiltration has been found to be lower in subjects with elevated RBM15B expression compared with patients with low RBM15B expression. (Figure 7B). At last, we looked at the differences between the two immune cell infiltration m^6^A patterns. Cluster A was shown to be linked with B-cells, CD8 T-cells, as well as dendritic cells, whereas cluster B was discovered to be related with macrophages and neutrophils (Figure 7C).

### Differentiation of two different m^6^A–related gene patterns and characteristics

**FIGURE 8:**
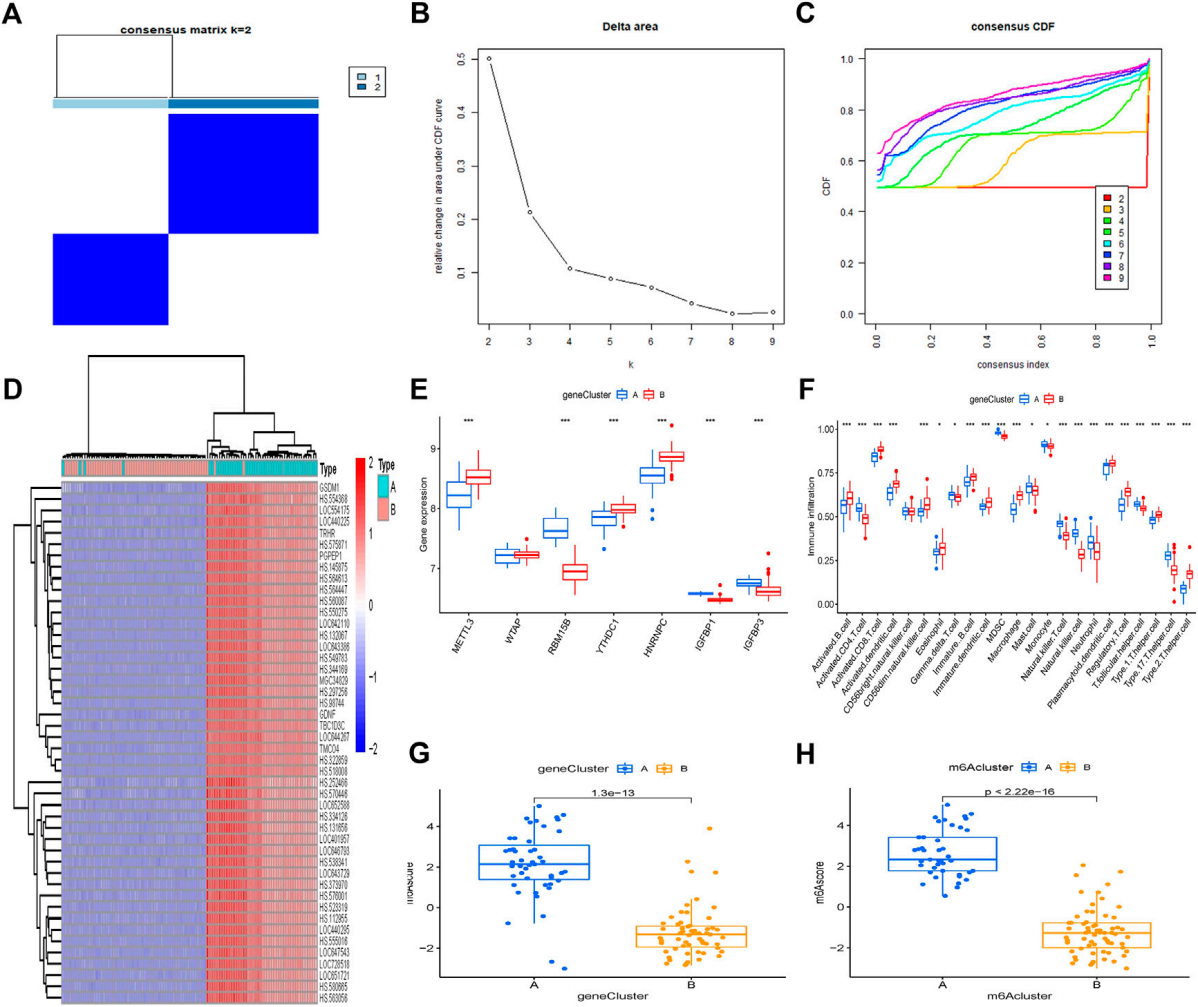
Clustering of the DEGs in two m^6^A clusters by consensus. **(A)** For *k* = 2, consensus matrices of the seven major m^6^A regulators. **(B)** The delta area difference for *k* = 2–9. **(C)** The CDF curve’s area under the curve. **(D)** Heatmap of DEG expression in gene clusters A and B. **(E)** Histogram showing differential expression of the seven major m^6^A regulators in gene clusters A and B. **(F)** Levels of immune cell infiltration in gene groups A and B. **(G)** M^6^A score differences between gene clusters A and B. **(H)** The m^6^A score differences between gene clusters A and B. *p < 0.05, **p < 0.01, and ***p < 0.001.

Using a consistent clustering algorithm, individuals with OA were categorized into several genetic subgroups based on m^6^A–associated DEGs. We discovered two distinct m^6^A–related gene clusters (gene group A and gene group B), which correspond to the m^6^A pattern grouping (Figure 8A). Figure 8B shows the delta area of the curve. When *k* = 2, the area under the curve tends to be stationary; thus, 2 is the most appropriate *k*-value. Figure 8C depicts the cumulative distribution function as *k* varies. When *k* = 2, the cumulative distribution function (CDF) achieves an approximate maximum, cluster analysis findings are the most trustworthy, and the resultant classification is the best. DEGs in gene clusters A and B have distinct degrees of expression (Figure 8D). Infiltration of immune cells between gene clusters A and B is comparable with that seen in the m^6^A pattern (Figures 8E, F). This confirms that the consensus clustering technique grouping is valid once again. When we compare the m^6^A scores of the two m^6^A patterns, we can observe that group A or gene group A has a higher m^6^A score than group B or group B (Figures 8G, H).

### Role of m^6^A patterns in distinguishing osteoarthritis

**FIGURE 9:**
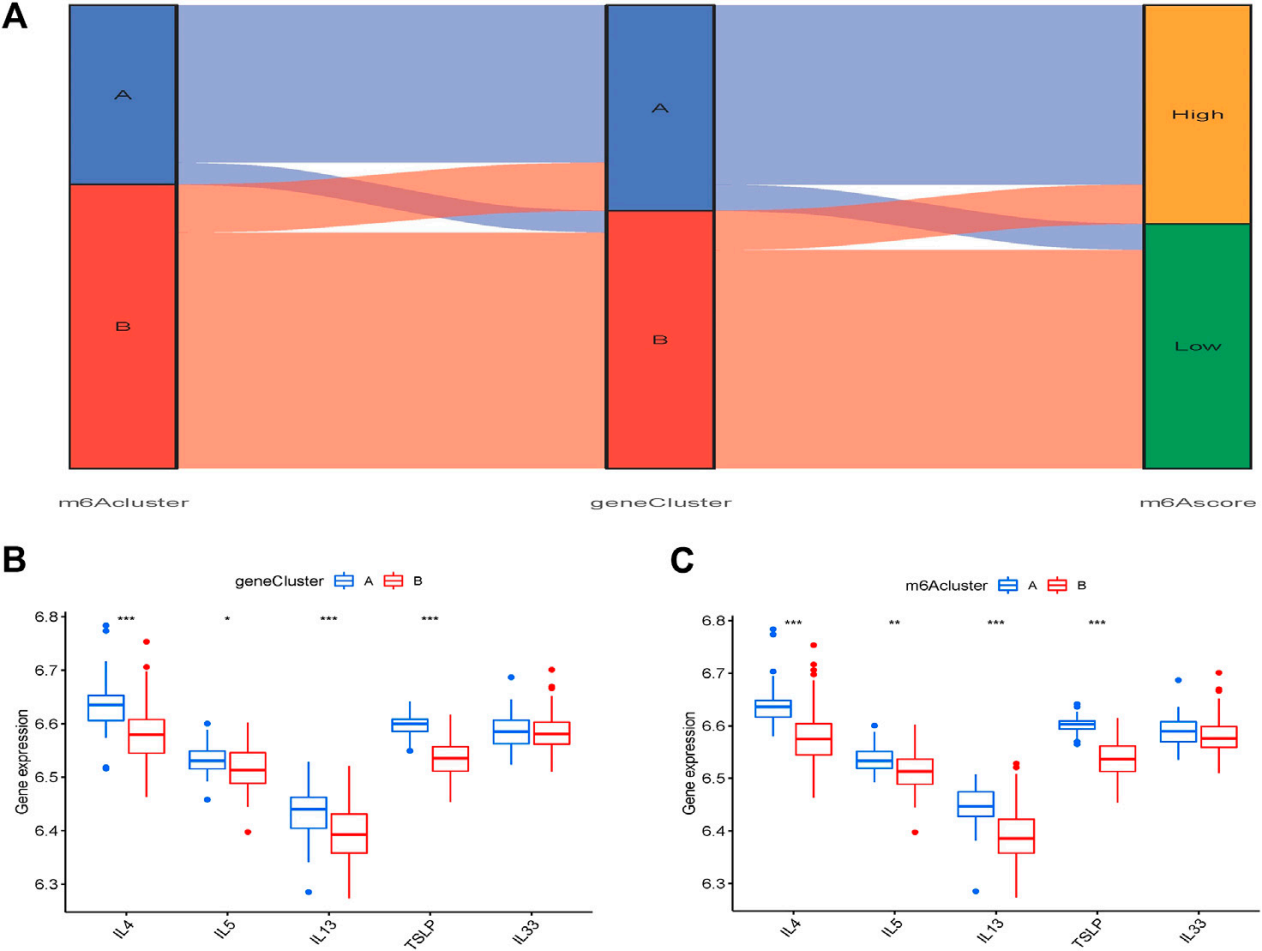
Influence of m^6^A pattern in OA. **(A)** A Sanchi plot illustrating the link between m^6^A pattern, m^6^A gene pattern, and m^6^A score. **(B)** Different transcriptional activity of thymic stromal IL-4, IL-5, IL-13, lymphopoietin (TSLP), and interleukin (IL)-33 clusters A and B. **(C)** Different transcriptional activity of IL-4, IL-5, IL-13, TSLP, and IL-33 between gene clusters A and B. *p < 0.05, **p < 0.01, and ***p < 0.001.

The Sankey plot depicts the association between the m^6^A pattern, the m^6^A gene pattern, and the m^6^A score (Figure 9A). IL-4, IL-5, IL-13, thymic stromal lymphopoietin (TSLP), and IL-33 were used to investigate the m^6^A pattern’s relationship to OA. The findings revealed that TSLP, IL-4, IL-5, and IL-13 expression levels in cluster A were considerably greater than those in cluster B (Figures 9B, C).

## Discussion

OA is a degenerative joint disease that most often affects the elderly. It is marked by gradual cartilage deterioration, as well as synovial membrane inflammation, osteophyte production, and subchondral bone sclerosis (Charlier et al., 2019). Although the m^6^A regulator has been linked to a variety of biological processes, its involvement in OA remains unknown. The purpose of our study was to see whether m^6^A regulators were involved in OA patients.

Through differential expression analysis in individuals with non-OA and OA, we initially identified seven significant m^6^A regulators out of 26 m^6^A regulators. To forecast the incidence of OA, an RF model was built, and seven prospective m^6^A regulators (IGFBP3, WTAP, IGFBP1, HNRNPC, RBM15B, YTHDC1, and METTL3) were chosen among 26 m^6^A regulators. Then, using seven candidate m^6^A regulators, a line chart model was created, and the DCA curve revealed that this column line chart model might be effective for patients with OA.

Among these seven important m^6^A regulatory factors, IGFBP1 and IGFBP3 are proteins that regulate the growth and proliferation of somatic cells which play a vital role in the secretion and role of growth hormone (Ranke, 2015). They can increase the stability of mRNA to regulate mRNA expression. It has been shown that IGFBP1 and IGFBP3 are associated with extensive extracellular matrix loss, cell death, and the formation of an inflammatory environment in terms of initiating the typical OA response pattern, providing new ideas for the assessment and treatment of OA (Schlichting et al., 2014). Another research identified chondrocyte enlargement as a detrimental event in the development of OA, which was significantly averted when chondrocytes were knocked out of IGFBP3 (Evans et al., 2015). WTAP is a nuclear protein that, like WT1, is found in speckles across the nucleoplasm and partly colocalizes with splicing factors (Little et al., 2000). WTAP may be found on nuclear plaques that are rich in pre-mRNA processing factors, which is essential for m^6^A methyltransferase catalytic activity *in vivo* and can also control the recruitment of the m^6^A methyltransferase complex to mRNA targets (Ping et al., 2014). HNRNPC (nuclear heterogeneous ribonucleoprotein C) is a widely expressed RNA-binding protein that aids in the shearing of introns and the proper assembly of exons during RNA splicing (Liu et al., 2015). It has been shown that the HNRNP family can cause degradation of the extracellular matrix and cartilage loss associated with it, which in turn can initiate OA response patterns (Ruedel et al., 2013). The WTAP-METTL3 m^6^A methyltransferase complex contains RBM15, which is an RNA-binding protein. WTAP interacts with RBM15B and related RBM15 to recruit complexes to target mRNAs and cleavage factors like as SF3B1 to promote selective shearing (Patil et al., 2016). YTHDC1 controls mRNA splicing by attracting and modifying pre-mRNA splicing factors, allowing them to reach specific mRNAs’ critical areas (Xiao et al., 2016). The best-known m^6^A methyltransferase, METTL3, is involved in the reversible epi-transcriptome control of m^6^A modification (Liu et al., 2020). Some studies have shown that intra-articular injection of synovially targeted METTL3 siRNA inhibits the propagation of cellular senescence in the joint and facilitates destabilisation of the medial meniscus (DMM)-induced cartilage destruction (Chen et al., 2022).

The complexity of OA creates obstacles to its effective treatment. At present, some studies have suggested that the intervention of these factors that affect the development and differentiation of Th17 cells, such as mTORC1/mTORC2, may lead to the regulation of OA (Ren et al., 2016; Dar et al., 2018). Some researchers now believe that adaptive immunity and OA are related (Geyer and Schonfeld, 2018). The adaptive immune response is the procedure through which antigen-specific T/B lymphocytes are activated, increased, and differentiated into effector cells in response to antigenic stimulation in the body, resulting in various biological consequences. T-lymphocytes (T-cells) orchestrate the elimination of infections, whereas B lymphocytes (B-cells) create antibodies (Woodell May and Sommerfeld, 2019). Type 2 helper T-cells and mastocytes both release IL-4. Chondrocytes revealed lower sensitivity to the effects of IL-4 in the course of OA, which might be responsible for the fast deterioration of the articular cartilage (Millward–Sadler et al., 1999). IL-13, similar to IL-4, is a mediator of the body’s immune response and is secreted by a variety of cells, such as CD4^+^ T-cells, CD8^+^ T-cells, mast cells, basophils, eosinophils, and natural killer cells. IL-13 can inhibit the proinflammatory effects of TNF alpha relative to fibroblasts from OA patients (Yeh et al., 1995; Hsieh et al., 2013). IL-5 modulates the transcription of genes encoding in B-cell proliferation, cell survival, maturation, and effector activities, as well as innate and acquired immunological responses and eosinophilia (Dougan et al., 2019). The cytokines TSLP and IL-33 signaling pathways are essential in various inflammatory and allergy reactions (Hong et al., 2020). We discovered two m^6^A groups based on seven critical m^6^A regulators using the consensus clustering approach. Type A shows a significant increase in natural killer cells and neutrophils. At the same time, there is also a significant increase in inflammatory factors, including IL4, Il5, and IL33. Type B showed significantly increased B-cells, CD8 T-cells, Th1, and Th2 cells. It is more suggested that in the two types, the innate immune response is more dominant in type A, and the adaptive immune response is more dominant in type B. Different immune response methods can better guide the clinical immunotherapy of OA. We subsequently used 639 m^6^A-associated DEGs to check the accuracy of these findings in the m^6^A gene pattern. At last, to measure the m^6^A design, we use the PCA approach to calculate the m^6^A score for each sample. Because OA is a progressive disease with different treatments at different stages, our study can distinguish patients with high scores of acquired immune response from gene methylation modification regulation. Can it provide a new idea for treating OA in the future?

## Conclusion

In conclusion, we established the prediction model of OA using the m^6^A regulatory factor. From the perspective of m^6^A modification, OA patients are classified to identify the population with a robust immune response, which provides a new idea for the phased inflammatory immunotherapy of OA diseases.

## Data Availability

The datasets used during the present study are available from GEO (https://www.ncbi.nlm.nih.gov/geo/) database, accession number GSE48556.

## Supplementary material

Supplementary Figure S1 Q–Q plot.

Supplementary File S1 26 m^6^A regulators.

Supplementary File S2 639 m^6^A–correlated DEGs.
