# Supplementary figures and images for: Significance of m6A regulatory factor in gene expression and immune function of osteoarthritis
Source: Front Physiol. 2022 Sep 8;13:918270. doi: 10.3389/fphys.2022.918270 (PMC9493330; doi:10.3389/fphys.2022.918270)

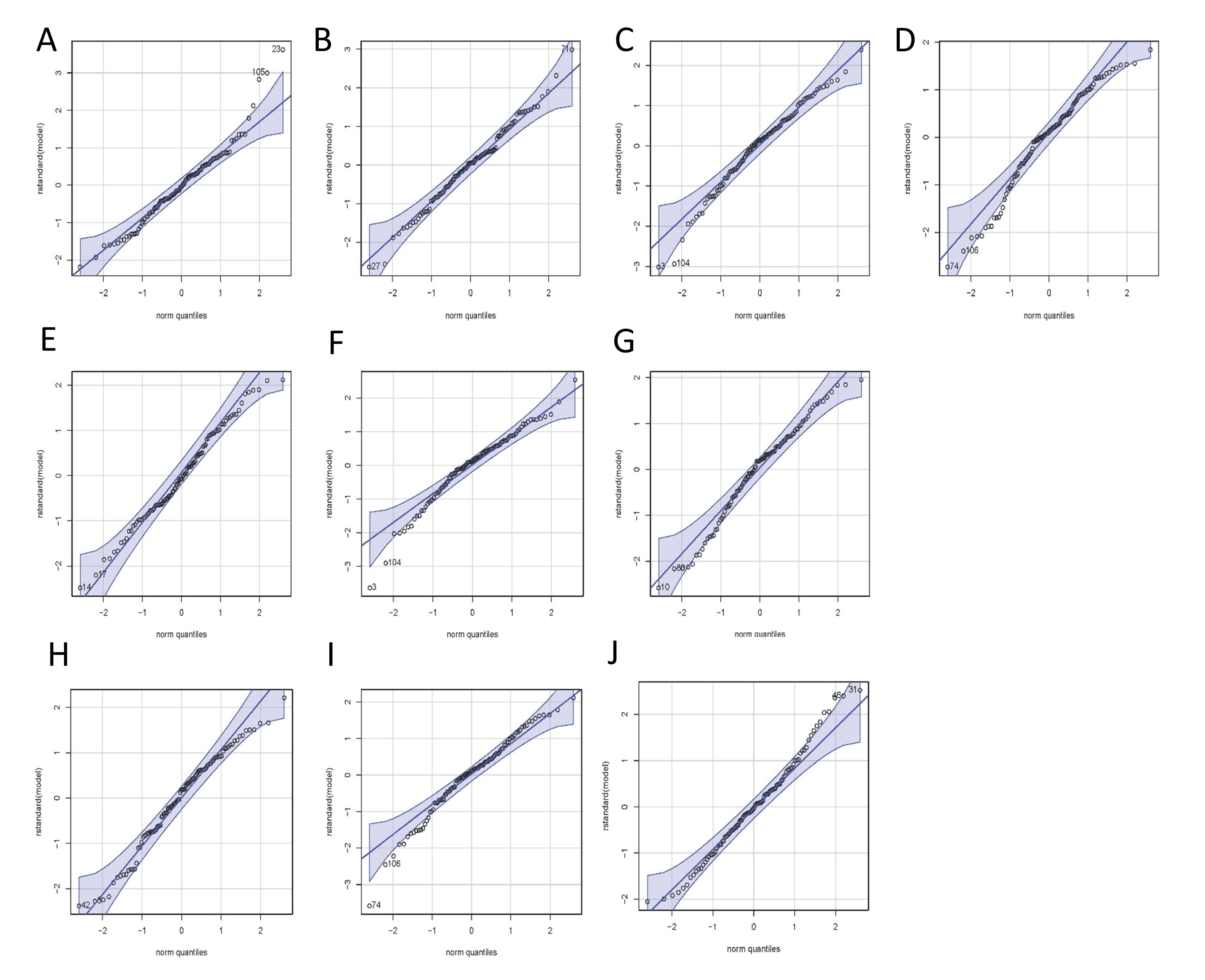

Supplement: Supplementary file 2 [file Image1.JPEG]
